# The impact of chronic electronic cigarette use on endothelial dysfunction measured by flow-mediated vasodilation: A systematic review and meta-analysis

**DOI:** 10.18332/tid/186932

**Published:** 2024-05-22

**Authors:** Jieun Lee, Zhiqi Yao, Ellen Boakye, Michael J. Blaha

**Affiliations:** 1 Department of Epidemiology, Johns Hopkins Bloomberg School of Public Health, Baltimore, United States; 2 Johns Hopkins Ciccarone Center for the Prevention of Cardiovascular Disease, Baltimore, United States

**Keywords:** endothelial dysfunction, e-cigarette, ENDS, flow-mediated vasodilation, vaping

## Abstract

**INTRODUCTION:**

Despite electronic cigarettes (e-cigarettes) being marketed as a safer alternative to combustible cigarettes, the effects of chronic e-cigarette use on vascular health remain uncertain. Our meta-analysis aimed to assess the health implications of chronic exclusive e-cigarette use on endothelial dysfunction, as measured by flow-mediated vasodilation (FMD).

**METHODS:**

PubMed, Embase and Scopus were searched for studies from 1 January 2004 to 31 March 2024. Four cross-sectional studies (n=769) were pooled using a random-effects model. The mean differences (MD) of FMD were reported by comparing exclusive e-cigarette use versus non-use; exclusive e-cigarette use versus combustible cigarette use; and combustible cigarette use versus non-use.

**RESULTS:**

A non-significant reduction in FMD in exclusive e-cigarette use compared to non-use was reported (MD of FMD: -1.47%; 95% CI: -3.96 – 1.02; I^2^= 84%). Similar MD of FMD in exclusive e-cigarette use and exclusive combustible cigarette use (vs non-use) suggested that both of these products might have comparable adverse influences on endothelial health.

**CONCLUSIONS:**

The limited availability of studies assessing the chronic impact of e-cigarette use restricted our ability to provide definitive findings. We emphasize the importance of additional research that explores the long-term impact of e-cigarette use on endothelial dysfunction, and identify key areas and give suggestions for further study.

## INTRODUCTION

The emergence of electronic cigarettes (e-cigarettes) has brought about a significant shift in tobacco consumption since their release around 2003. Marketed as a purportedly safer alternative to combustible cigarettes, e-cigarettes have gained substantial popularity, particularly among youth and former smokers. A recent study estimated that 10.8 million adults were using e-cigarettes in 2016^1^, with past 30-day e-cigarette use among high school students rising from 1.5% in 2011 to 11.7% in 2017, and further to 20.8% in 2018 in the US^2^.

Endothelial dysfunction, an early marker and independent prognostic predictor for cardiovascular diseases (CVD) risk, is one potential adverse consequence of e-cigarettes^3^. It is characterized by impaired endothelium-mediated vasodilation, reduced nitric oxide levels, increased inflammatory response, and a prothrombotic state^4^. When endothelial damage occurs alongside decreased nitric oxide availability, the ability for physiological vasodilation diminishes, leading to acute coronary events and atherothrombosis^5^. It has been reported that e-cigarettes may activate nicotinamide adenine dinucleotide phosphate oxidase and disrupt endothelial nitric oxide synthase (eNOS) activation and coupling, leading to a vicious cycle of superoxide generation, peroxynitrite formation, and tetrahydrobiopterin depletion, causing loss of nitric oxide (NO) and triggering vascular endothelial dysfunction^6^.

Previous research highlighted the potential harmful effects of immediate impact of e-cigarette aerosol exposure on endothelial dysfunction, measured by flow-mediated vasodilation (FMD), the most commonly used non-invasive method for assessing endothelial function^7-9^. A systematic review focusing on the acute effects of e-cigarettes in crossover studies demonstrated that both nicotine-containing and nicotine-free e-cigarettes significantly decrease FMD, indicating impaired endothelial function^10^. For instance, Carnevale et al.^11^ reported that a substantial acute decrease in FMD following a single session of e-cigarette use. Furthermore, Ben et al.^12^ suggested a decline in peripheral macrovascular function, as assessed through FMD, after taking 10 puffs of e-cigarettes. However, it remains unclear whether these acute changes translate into long-term impacts on endothelial health, as a substantial portion of the existing data originated from preclinical studies^13-15^.

Therefore, this study aims to pool all studies evaluating the chronic impact of exclusive e-cigarette exposure on endothelial dysfunction measured by FMD to provide users and healthcare systems with better insight into their potential association with a crucial early marker of CVD^10^.

## METHODS

### Data sources and search strategies

This systematic review and meta-analysis was conducted according to the Preferred Reporting Item for Systematic Reviews and Meta-analysis (PRISMA) 2020 guidelines (Supplementary file Table 1)^16^. The review was not registered with PROSPERO. Eligible studies were drawn from an electronic search of PubMed, Embase, and Scopus from 1 January 2004 to 31 March 2024. The search strings used are provided in Supplementary file Table 2. Studies were also identified by manually searching the references of published articles, reviews, and previous meta-analysis. Literature search, study selection, and data extraction were performed by two individuals separately (JL, ZY).

### Study selection

The studies were included if they met all of the following criteria: 1) published observational studies; 2) measured FMD data at baseline; 3) had exclusive e-cigarette use data or adjusted results for possible confounding factors (e.g. combustible cigarette use); and 4) written in English. All animal studies, *in vivo* or *in vitro* studies, studies only reporting acute exposure to e-cigarettes, and review articles were excluded.

### Data extraction and quality assessment of studies

Articles retrieved from the search were exported to Zotero (Corporation for Digital Scholarship, VA) and screened for duplicates. Titles and abstracts were then screened by two independent reviewers (JL, ZY) for abstract and full-text assessment against the inclusion criteria for the review. Any disagreements between the reviewers at each stage of the study selection process were resolved through discussion. In assessing the quality of non-randomized studies for this meta-analysis, the Newcastle-Ottawa Scale was utilized^17^. This scale comprises seven items categorized into three subscales: selection, comparability, and outcome. Two reviewers (JL, ZY) independently scored the studies, and any discrepancies were resolved through consensus. Each study could attain a maximum score of 10: 9–10 (very good), 7–8 (good), 5–6 (satisfactory), and 0–4 (unsatisfactory). Funnel plots and Egger’s regression test were used to assess publication bias in the pooled MD of FMD^18^.

### Statistical analysis

The mean differences (MD) of FMD, with 95% confidence intervals (CI), are reported by comparing exclusive e-cigarette use versus non-use; exclusive e-cigarette use versus combustible cigarette use; and combustible cigarette use versus non-use. MD was computed instead of standardized mean differences, as all FMD data across studies used the same unit of percent^19^. To pool the data, a random-effects model was employed. Higgins’ I^2^ test was used to explore heterogeneity. A value of I^2^=25–50% was considered mild, 50–75% as moderate, and >75% as severe^10,20^. A two-sided p<0.05 was considered statistically significant. Stata version 17 was used for all statistical analyses.

## RESULTS

### Literature search

**Figure 1:**
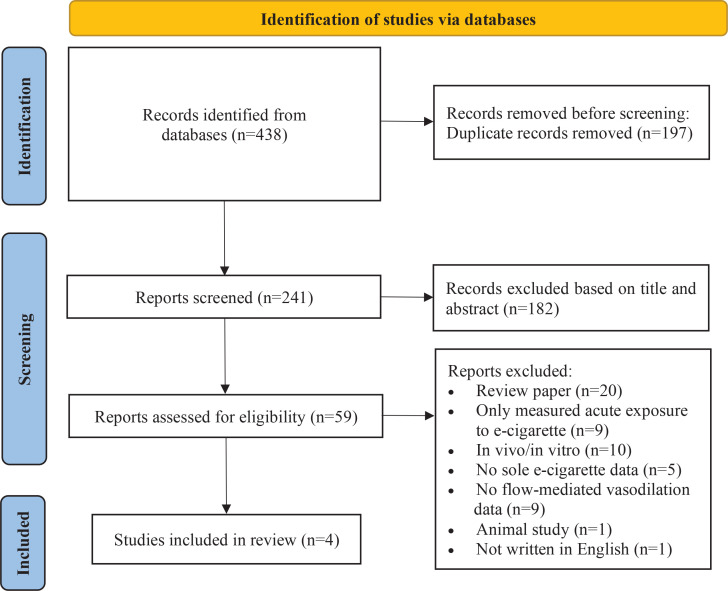
PRISMA flow chart

The initial search yielded 438 articles. After screening for duplicates, 241 remained. A total of 182 studies were excluded based on title and abstract; 59 were assessed for eligibility and 55 articles were excluded, leaving total of 4 articles for the meta-analysis (Figure 1). Supplementary file Table 3 provides exclusion reasons for articles assessed for eligibility.

### Study characteristics

All four included studies in this meta-analysis are cross-sectional studies conducted in the US, with Haptonstall et al.21 adding a randomized crossover study to evaluate immediate alterations in FMD following e-cigarette use^21-24^. The participants (n=769) were young and healthy individuals who were free of CVD and traditional cardiovascular risk factors.

A total of 150 individuals were categorized as exclusive e-cigarette users, defined as those who had either never used combustible cigarettes or had quit prior to the study, 353 were exclusive combustible cigarette users, and 214 were non-users. FMD measurements were only available with 648 participants (exclusive e-cigarette use n=130; exclusive combustible use n=338; and non-use n=180). These studies used varied generations of e-cigarettes, ranging from the first generation to the fourth. For FMD measurement, all four studies induced forearm occlusion for 5 minutes with the cuff inflated to 250 mmHg, except for one study, which inflated the cuff to either 200 mmHg or 50 mmHg higher than the systolic blood pressure. Different automatic software for brachial artery measurement was utilized across the selected studies, with two studies sharing the same software (Vascular Analysis Tools, Medical Imaging Applications, LLC) (Supplementary file Table 4). To minimize the influence of other acute factors on FMD, all studies required participants to abstain from specific behaviors (e.g. smoking, caffeine, exercise, vaping, food, and alcohol consumption) for at least 8 hours before the study visit. Detailed information on the studies is presented in Table 1.

**Table 1:** Overview of four selected studies for meta-analysis (N=769)

*Authors Year*	*Country*	*Study design*	*Total*	*Study participants*	*Number of EC with FMD data*
Haptonstall et al.^21^ 2020	US	Cross-sectional, Randomized crossover	136 47 NU 49 EC 40 CC	Males and females, aged 21–45 years, free of CVD	49
Mohammadi et al.^22^ 2022	US	Cross-sectional	120 50 NU 42 EC 28 CC	Males and females, aged 21–50 years, free of CVD	22
Fetterman et al.^23^ 2020	US	Cross-sectional	467 94 NU 36 EC 285 CC 52 DU	Males and females, aged 21–45 years, free of CVD	36
Boakye et al.^24^ 2023	US	Cross-sectional	46 23 NU 23 EC	Males and females, aged 18–34 years, free of CVD	23
***Age of EC (years) Mean ± SD***	***Sex distribution of EC***	***Definition of exclusive EC***	***Method to minimize acute effect***	***Measured vascular dysfunctionrelated outcomes***	***Type of e-cigarettes***
27.2 ± 5.7	67.7% male	Chronic (>12 months) e-cig use, quit tobacco smoking >1 year	Smoking, caffeine, exercise abstinence 12 hours before the study visit	FMD, velocity reactive hyperemia, shear stress reactive hyperemia	2nd generation, JUUL
29 ± 4.6	76.2% male	Current use of e-cig (>5 times/week for >3 months), no current use of tobacco products	Vaping, smoking, coffee abstinence 12 hours before the study visit	FMD, NO production, NOS3 gene, eNOS level, H2O2 in cell culture supernatant, lysed cells and serum, endothelial cell permeability, circulating biomarkers (RAGE, HMGB1, S100A8 gene, vWF, E-selectin gene, IL-1 β, IFN- β, MPO, PECAM-1, sICAM -1)	Mainly 1st–3rd generation
29 ± 6	72% male	Current use of e-cig (≥5 days/week), quit tobacco smoking ≥3 months	Food, tobacco product abstinence 8–12 hours before the study visit	FMD, flow velocity, hyperemic flow velocity, hyperemic shear stress, NO production, eNOS level, carotidfemoral PWV, carotid-radial PWV, AIx	2nd, 3rd generation
23.0 ± 3.7	78.3% male	Current use of e-cig (≥6 consecutive months), no current use of tobacco products	Food abstinence 10 hours, caffeine, alcohol abstinence 24 hours before the study visit	FMD, reactive hyperemia index, high-sensitivity C-reactive protein, interleukin-6, fibrinogen, P-selectin, myeloperoxidase	Rechargeable, refillable, modular

NU: non-use. EC: exclusive e-cigarette use. CC: exclusive combustible cigarette use. DU: dual use. CVD: cardiovascular disease. FMD: flow-mediated vasodilation. NO: nitric oxide. NOS3: nitric oxide synthase 3. eNOS: endothelial nitric oxide synthase. H_2_O_2_: hydrogen peroxide. RAGE: receptor for advanced glycation end products. HMGB1: high mobility group box 1. vWF: von Willebrand factor. IL-1β: interleukin-1β. IFN-β: interferon-β. MPO: Myeloperoxidase. PECAM-1: platelet endothelial cell adhesion molecule-1. sLCAM-1: soluble intercellular adhesion molecule-1. PWV: pulse wave velocity. AIx: augmentation index.

All included studies had good or satisfactory risk of bias, scoring 6 or 7 out of 10 points on the Newcastle-Ottawa Scale (Supplementary file Table 5). We could not conclude on the presence of asymmetry in the funnel plots used to evaluate publication bias in the pooled MD of FMD due to the small number of selected studies for meta-analysis (Supplementary file Figures 1–3)^18^. However, Egger’s regression test did not reveal any significant publication bias when comparing exclusive e-cigarette use versus non-use; exclusive e-cigarette use versus combustible cigarette use; and combustible cigarette use versus non-use (p=0.288, 0.194, 0.236, respectively).

### Results of the meta-analysis: FMD comparison

**Figure 2:**
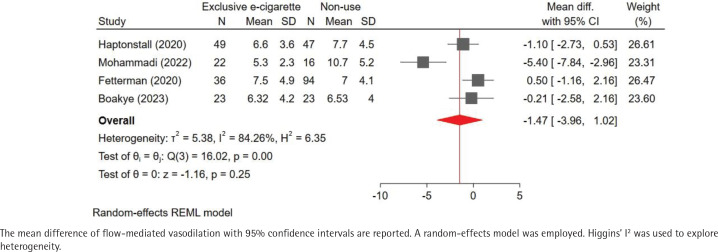
Pooled result of the mean difference of flow-mediated vasodilation (exclusive e-cigarette use vs nonuse)

**Figure 3:**
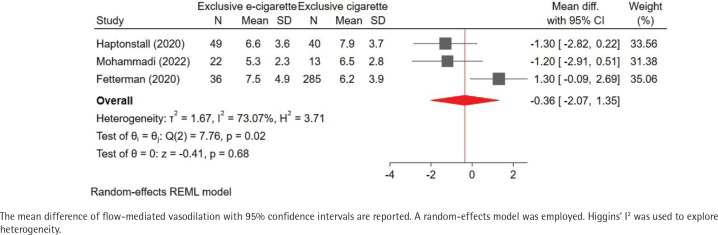
Pooled result of the mean difference of flow-mediated vasodilation (exclusive e-cigarette use vs exclusive combustible cigarette use)

**Figure 4:**
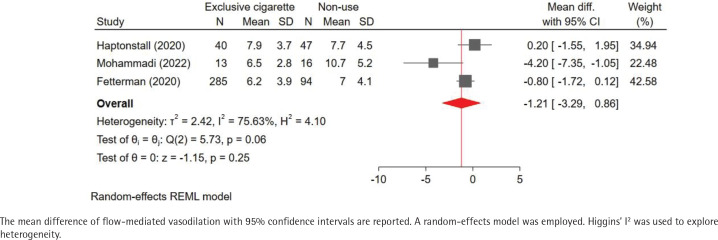
Pooled result of the mean difference of flow-mediated vasodilation (exclusive combustible cigarette use vs non-use)

Our pooled analysis indicated that exclusive e-cigarette use may reduce FMD compared to non-use, although this effect was not statistically significant (MD of FMD: -1.47%; 95% CI: -3.96–1.02; I^2^= 84%) ( Figure 2). Comparison of FMD with exclusive combustible cigarette use was available for only three studies. Notably, FMD was slightly lower in exclusive e-cigarette use compared to exclusive combustible cigarette use (MD of FMD: -0.36%; 95% CI: -2.07–1.35; I^2^=73%) (Figure 3). Furthermore, the MD of FMD for exclusive combustible cigarette use versus non-use was similar to that for exclusive e-cigarette use versus non-use, suggesting that both of these products might have comparable adverse influences on endothelial health [MD of FMD (exclusive combustible cigarette use vs non-use): -1.21%; 95% CI: -3.29–0.86; I^2^= 76%; Figure 4].

### Comparison of other biomarkers of endothelial dysfunction

Fetterman et al.^23^ found no significant difference in FMD levels among exclusive e-cigarette users but explored other markers of endothelial dysfunction, including augmentation index (Alx), eNOS, and A23187-induced NO. The Alx indicated a similar detrimental impact on arterial stiffness between exclusive e-cigarette use and exclusive combustible cigarette use after adjusting for potential confounders (126.2 ± 5.9 vs 129.8 ± 1.5; p=1.0). Notably, exclusive e-cigarette use exhibited lower levels of eNOS compared to exclusive combustible cigarette use (10.7 ± 2.2 arbitrary units vs 22.1 ± 3.6 arbitrary units; p=0.03). Reduced NO production was observed in exclusive e-cigarette use compared to non-use (2.6 ± 3.0% vs 14.1 ± 1.5%; p=0.018). This reduction was also comparable between exclusive e-cigarette use and exclusive combustible cigarette use (p=0.828), signifying a similar impairment in NO signaling.

## DISCUSSION

Our meta-analysis found that chronic exclusive exposure to e-cigarettes may potentially pose a small but meaningful long-term detrimental effect on endothelial health. This is evident from the numerical but non-significant decrease in FMD levels, as well as reduced NO production and eNOS levels in exclusive e-cigarette use. Furthermore, the augmented levels of Alx were observed among exclusive e-cigarette users, signaling the potential emergence of hypercholesterolemia and hypertension^25^. These findings indicate a potential impairment of endothelial function of exclusive e-cigarette use, which in turn can contribute to the development of atherosclerosis, hypertension, and other CVD^4^.

While there are no universally standardized values for diagnosing endothelial dysfunction through FMD, Maruhashi et al.^26^ suggested an optimal cutoff value of 7.1% to effectively distinguish individuals with CVD risk factors. Notably, the FMD values for exclusive e-cigarette use in three selected studies were found to be below the proposed diagnostic threshold of 7.1%, indicating a potential state of dysfunction^21,22,24^. Mohammadi et al.^22^ demonstrated a statistically significant difference in the FMD between exclusive e-cigarette use compared to non-use (5.3 ± 2.3% vs 10.7 ± 5.2%; p<0.001). Both Haptonstall et al.^21^ and Boakye et al.^24^ indicated lower FMD levels in exclusive e-cigarette use compared to non-use, although these outcomes did not reach statistical significance (6.6 ± 3.6% vs 7.7 ± 4.5%; p=0.35, and 6.32 ± 4.15% vs 6.53±3.98%; p=0.58, respectively). Despite the limited sample size, the relatively consistent observation of lower FMD levels in exclusive e-cigarette use implies the potential for chronic e-cigarette use to have an adverse impact on FMD over the long-term, thus justifying our meta-analysis.

Endothelial dysfunction, an initial reversible stage in atherosclerosis development, has emerged as both an important biomarker and a potential therapeutic target^27^. As endothelial function becomes impaired, the restoration of vascular function may be achieved by mitigating or eliminating risk factors^28^. Therefore, the early detection of potential CVD risks associated with e-cigarettes through endothelial dysfunction is crucial, as it serves as an early indicator of the development of atherosclerosis and subsequent risk of atherosclerotic CVD over the long-term^29^. This focus on endothelial dysfunction is vital for guiding regulatory bodies, such as the Food and Drug Administration (FDA), in designing effective regulations and product standards that safeguard the health of consumers and future users, making it a pivotal element in assessing the impact of e-cigarettes on vascular health.

### Limitations

Our study has certain limitations. First, though a trend has been seen across studies, the limited number of selected observational studies and small sample sizes measuring endothelial function in exclusive e-cigarette use restricted our ability to establish statistically significant differences in FMD between e-cigarette use and non-use. For our observed MD of -1.47% in FMD to be statistically significant, we have calculated that at 90% power, the required minimum sample size would be approximately 328 (164 in the exclusive e-cigarette group, and 164 in the non-use group). After excluding the study conducted by Mohammadi et al.^22^, which exhibited the largest FMD difference, the required minimum sample size would be 4520 (2260 in the exclusive e-cigarette group, and 2260 in the non-use group) with a MD of -0.29% in FMD and a power of 80%.

Second, the variability in methodologies for measuring FMD also may limit the reliability of pooled effect sizes, as differences in occlusion cuff position and protocol could yield disparate results^30^. For instance, while all studies induced forearm occlusion, the cuff was placed at different distances from the antecubital fossa across studies (e.g. just below, 1 cm distal to, or 2 cm above), potentially influencing outcomes. Furthermore, inconsistent occlusion pressure among studies may impact FMD values, as higher pressure can inflate results, particularly in small studies^31^.

Third, FMD is not a gold standard for endothelial functional assessment, which is invasive quantitative angiography^32^, potentially raising concerns about validation (e.g. learning curve)^33^. However, previous literature highlighted FMD as a relatively accurate method for measuring CVD risks, exhibiting relatively good sensitivity, specificity, and reproducibility, which helps alleviate such concerns^34-36^.

Fourth, the definition of exclusive e-cigarette use varies among the selected studies regarding the frequencies and duration of e-cigarette exposure, which limits our ability to estimate pooled results for specific usage patterns. Moreover, while two studies classified participants as ‘exclusive’ e-cigarette users based on the duration since quitting combustible cigarette use (e.g. more than 1 year or 3 months of cessation), the remaining two studies define them as individuals who currently do not use tobacco products. This discrepancy raises concerns about the potential lasting effects of combustible cigarettes, as quitting smoking does not immediately diminish the risk of CVD^37^.

Finally, a wide array of e-cigarette types and generations, ranging from 1st generation (cig-a-like) to 4th generation (pod-based e-cigarettes, e.g. JUUL), with different ingredients and delivery systems prevented our ability to conduct a comprehensive review that encompassed the entire spectrum of e-cigarette usage^38,39^. This scarcity of published studies focusing on the impact of chronic e-cigarette use and the limited scope of research underscores the urgent need for further research to bridge this gap in knowledge.

### Further research required for assessing long-term influence of e-cigarettes on endothelial dysfunction

A more comprehensive range of investigations with adequately powered sample sizes and longer follow-up, is necessary to assess the long-term influence of e-cigarette use on endothelial dysfunction and CVD. First, studies should be conducted to explore the distinct impacts of e-cigarette use across different use patterns, exposure durations, and frequencies. Second, given the evolving landscape of e-cigarettes, it is essential to explore the effects of different e-cigarette devices on cardiovascular health. Notably, the disposable e-cigarettes, preferred by youth for their efficient nicotine delivery, enticing flavors, and sleek designs, require focused studies due to their popularity^40,41^. Third, research should address the impact of various nicotine levels in e-cigarettes. Especially, since nicotine-free e-cigarettes, which are often marketed as harmless alternatives, have exhibited adverse acute effects on endothelial function, achieving consensus on this is pivotal^42^. Lastly, further studies are needed to isolate the independent impact of e-cigarette use on cardiovascular health for exclusive users, as a considerable proportion of e-cigarette users previously engaged in smoking cigarettes. The lingering effects from prior combustible cigarette use could potentially confound endothelial function measurements, complicating the establishment of a true relationship.

In light of these pressing research needs, we implore relevant stakeholders, including government agencies, public health organizations, healthcare professionals, and the private sector, to prioritize this area of research and provide the necessary funding needed to conduct adequately powered longitudinal studies, with sample sizes guided by our power calculations above. It will enable researchers to undertake these critical studies, advance our knowledge in this field, and guide informed decision-making, especially in guiding policies that will be appropriate for the protection of public health. The collective commitment to this research is a critical investment in the vascular health of current and future generations.

## CONCLUSIONS

While our meta-analysis demonstrated a potentially adverse health impact on endothelial dysfunction measured by FMD among exclusive e-cigarette users, the scarcity of relevant studies restricted our ability to establish a definitive association. Given that endothelial dysfunction serves as an early indicator of future CVD, additional funding is imperative to facilitate appropriately powered longitudinal studies examining the influence of e-cigarettes on endothelial function. We would like to emphasize the significance of FMD as a validated method of assessing endothelial function and identifying individuals who may be at an elevated risk of CVD^43^. The importance of this research lies in its potential to advance our understanding of the health implications of chronic e-cigarette use for current and future generations.

## Supplementary Material



## Data Availability

The data supporting this research are available from the authors on reasonable request.
