# Supplementary material for: The impact of chronic electronic cigarette use on endothelial dysfunction measured by flow-mediated vasodilation: A systematic review and meta-analysis
Source: Tob Induc Dis. 2024 May 22;22:10.18332/tid/186932. doi: 10.18332/tid/186932 (PMC11110651; doi:10.18332/tid/186932)
Supplement: Supplementary file 1 [file TID-22-84-s1.pdf]

Supplementary Table 1. PRISMA 2020 checklist

Topic	No.	Item	Location where item is reported
**TITLE**			
**Title**	1	Identify the report as a systematic review.	Title
**ABSTRACT**			
**Abstract**	2	See the PRISMA 2020 for Abstracts checklist	Abstract
**INTRODUCTION**			
**Rationale**	3	Describe the rationale for the review in the context of existing knowledge.	Introduction
**Objectives**	4	Provide an explicit statement of the objective(s) or question(s) the review addresses.	Introduction
**METHODS**			
**Eligibility criteria**	5	Specify the inclusion and exclusion criteria for the review and how studies were grouped for the syntheses.	Methods, Study Selection
**Information sources**	6	Specify all databases, registers, websites, organisations, reference lists and other sources searched or consulted to identify studies. Specify the date when each source was last searched or consulted.	Methods, Data Sources and Search Strategies
**Search strategy**	7	Present the full search strategies for all databases, registers and websites, including any filters and limits used.	Methods, Data Sources and Search Strategies

Topic	No.	Item	Location where item is reported
**Selection process**	8	Specify the methods used to decide whether a study met the inclusion criteria of the review, including how many reviewers screened each record and each report retrieved, whether they worked independently, and if applicable, details of automation tools used in the process.	Methods, Data Extraction and Quality Assessment of Studies
**Data collection process**	9	Specify the methods used to collect data from reports, including how many reviewers collected data from each report, whether they worked independently, any processes for obtaining or confirming data from study investigators, and if applicable, details of automation tools used in the process.	Methods, Data Extraction and Quality Assessment of Studies
**Data items**	10a	List and define all outcomes for which data were sought. Specify whether all results that were compatible with each outcome domain in each study were sought (e.g. for all measures, time points, analyses), and if not, the methods used to decide which results to collect.	Methods, Statistical Analysis
	10b	List and define all other variables for which data were sought (e.g. participant and intervention characteristics, funding sources). Describe any assumptions made about any missing or unclear information.	Methods, Statistical Analysis

Topic	No.	Item	Location where item is reported
**Study risk of bias assessment**	11	Specify the methods used to assess risk of bias in the included studies, including details of the tool(s) used, how many reviewers assessed each study and whether they worked independently, and if applicable, details of automation tools used in the process.	Methods, Data Extraction and Quality Assessment of Studies
**Effect measures**	12	Specify for each outcome the effect measure(s) (e.g. risk ratio, mean difference) used in the synthesis or presentation of results.	Methods, Statistical Analysis
**Synthesis methods**	13a	Describe the processes used to decide which studies were eligible for each synthesis (e.g. tabulating the study intervention characteristics and comparing against the planned groups for each synthesis (item 5)).	Methods, Statistical Analysis
	13b	Describe any methods required to prepare the data for presentation or synthesis, such as handling of missing summary statistics, or data conversions.	Methods, Statistical Analysis
	13c	Describe any methods used to tabulate or visually display results of individual studies and syntheses.	Methods, Statistical Analysis
	13d	Describe any methods used to synthesize results and provide a rationale for the choice(s). If meta-analysis was performed, describe the model(s), method(s) to identify the presence and extent of statistical heterogeneity, and software package(s) used.	Methods, Statistical Analysis

Topic	No.	Item	Location where item is reported
	13e	Describe any methods used to explore possible causes of heterogeneity among study results (e.g. subgroup analysis, meta-regression).	Methods, Statistical Analysis
	13f	Describe any sensitivity analyses conducted to assess robustness of the synthesized results.	Discussion, Limitations
**Reporting bias assessment**	14	Describe any methods used to assess risk of bias due to missing results in a synthesis (arising from reporting biases).	Methods, Data Extraction and Quality Assessment of Studies
**Certainty assessment**	15	Describe any methods used to assess certainty (or confidence) in the body of evidence for an outcome.	Methods, Data Extraction and Quality Assessment of Studies
**RESULTS**			
**Study selection**	16a	Describe the results of the search and selection process, from the number of records identified in the search to the number of studies included in the review, ideally using a flow diagram.	Results, Literature Search
	16b	Cite studies that might appear to meet the inclusion criteria, but which were excluded, and explain why they were excluded.	Results, Literature Search
**Study characteristics**	17	Cite each included study and present its characteristics.	Results, Study Characteristics
**Risk of bias in studies**	18	Present assessments of risk of bias for each included study.	Results, Study Characteristics

Topic	No.	Item	Location where item is reported
**Results of individual studies**	19	For all outcomes, present, for each study: (a) summary statistics for each group (where appropriate) and (b) an effect estimate and its precision (e.g. confidence/credible interval), ideally using structured tables or plots.	Results, Results of the Meta-analysis: FMD Comparison; Comparison of Other Biomarkers of Endothelial Dysfunction
**Results of syntheses**	20a	For each synthesis, briefly summarise the characteristics and risk of bias among contributing studies.	Results, Results of the Meta-analysis: FMD Comparison; Discussion, Limitations
	20b	Present results of all statistical syntheses conducted. If meta-analysis was done, present for each the summary estimate and its precision (e.g. confidence/credible interval) and measures of statistical heterogeneity. If comparing groups, describe the direction of the effect.	Results, Results of the Meta-analysis: FMD Comparison
	20c	Present results of all investigations of possible causes of heterogeneity among study results.	Results, Results of the Meta-analysis: FMD Comparison
	20d	Present results of all sensitivity analyses conducted to assess the robustness of the synthesized results.	Discussion, Limitations
**Reporting biases**	21	Present assessments of risk of bias due to missing results (arising from reporting biases) for each synthesis assessed.	Results, Study Characteristics

Topic	No.	Item	Location where item is reported
**Certainty of evidence**	22	Present assessments of certainty (or confidence) in the body of evidence for each outcome assessed.	Results, Study Characteristics
**DISCUSSION**			
**Discussion**	23a	Provide a general interpretation of the results in the context of other evidence.	Discussion
	23b	Discuss any limitations of the evidence included in the review.	Discussion, Limitations
	23c	Discuss any limitations of the review processes used.	Discussion, Limitations
	23d	Discuss implications of the results for practice, policy, and future research.	Discussion, Further Research Required for Assessing Long-term Influence of E-cigarettes on Endothelial Dysfunction
**OTHER INFORMATION**			
**Registration and protocol**	24a	Provide registration information for the review, including register name and registration number, or state that the review was not registered.	Methods, Data Sources and Search Strategies
	24b	Indicate where the review protocol can be accessed, or state that a protocol was not prepared.	Methods, Data Sources and Search Strategies

Topic	No.	Item	Location where item is reported
	24c	Describe and explain any amendments to information provided at registration or in the protocol.	Methods, Data Sources and Search Strategies
**Support**	25	Describe sources of financial or non-financial support for the review, and the role of the funders or sponsors in the review.	Funding
**Competing interests**	26	Declare any competing interests of review authors.	Conflicts of Interest
**Availability of data, code and other materials**	27	Report which of the following are publicly available and where they can be found: template data collection forms; data extracted from included studies; data used for all analyses; analytic code; any other materials used in the review.	N/A

Supplementary Table 2. Search string used for data extraction from ## to ##

**Database**	**Search string**
PubMed	((“E-Cigarette Vapor” [mesh] OR “Electronic Nicotine Delivery Systems” [mesh] OR “Vaping” [mesh] OR “e-cig*” OR “electronic cigarette” [tw] OR “Juul” [tw]) AND (“Flow-mediated dilation” [tw] OR “FMD” [tw] OR “forearm ischemia” [tw] OR “brachial artery dilation” [tw] OR “endothelial dysfunction” [tw] OR “Nitric Oxide Synthase” [mesh] OR “Nitric Oxide Synthase Type III” [mesh] OR “Hyperemia” [mesh] OR “Post-occlusive reactive hyperemia” [tw] OR “PORH” [tw]))
Embase	('electronic cigarette'/exp OR 'electronic cigarette' OR 'vaping'/exp OR vaping OR 'electronic cigarette vapor'/exp OR 'electronic cigarette vapor' OR 'juul'/exp OR juul) AND ('flow-mediated dilation test' OR 'flow mediated dilation' OR 'forearm ischemia' OR 'endothelial dysfunction' OR 'nitric oxide synthase' OR 'endothelial nitric oxide synthase' OR hyperemia OR 'post occlusive reactive hyperemia')
Scopus	(TITLE-ABS-KEY ("e-cigarette vapor" OR "electronic nicotine delivery systems" OR "vaping" OR "e-cig" OR "electronic cigarette" OR "juul") AND TITLE-ABS-KEY ("flow-mediated dilation" OR "fmd" OR "brachial artery dilation" OR "endothelial dysfunction" OR "nitric oxide synthase" OR "nitric oxide synthase type iii" OR "no synthase" OR "hyperemia" OR "post-occlusive reactive hyperemia" OR porh))

Supplementary Table 3. Reasons for exclusion in meta-analysis for papers assessed for eligibility (n= 55)

**First author**	**Publication year**	**Title**	**Reasons for exclusion**
Sheth	2024	The Rising Use of E-Cigarettes: Unveiling the Health Risks and Controversies	Review paper
Zong	2024	Electronic cigarettes and cardiovascular disease: epidemiological and biological links	Review paper
Sachdeva	2023	Flavoring Agents in E-cigarette Liquids: A Comprehensive Analysis of Multiple Health Risks	Review paper
Daiber	2023	E-cigarette effects on vascular function in animals and humans	Review paper
Spoladore	2022	The point on the electronic cigarette more than 10 years after its introduction	Review paper
Conklin	2022	How Irritating! Electronic Cigarettes Not "95% Safer" Than Combustible Cigarettes: Recent Mechanistic Insights Into Endothelial Dysfunction	Review paper
Han	2023	Impairment of Endothelial Function by Cigarette Smoke and e-Cigarette Aerosol Requires RAGE	Review paper
D'Amario	2019	Electronic cigarettes and cardiovascular risk: Caution waiting for evidence	Review paper
Groner	2022	Health effects of electronic cigarettes	Review paper
Kourea	2021	Mid-term effects of electronic cigarette use on vascular function and oxidative stress	Review paper
Kostelli	2020	Effects of combustible tobacco smoking and novel tobacco products on oxidative stress: Different sides of the same coin?	Review paper
Halstead	2023	Sex Differences in Oxidative Stress-Mediated Reductions in Microvascular Endothelial Function in Young Adult e-Cigarette Users	Review paper
Munzel	2023	Are e-cigarettes dangerous or do they boost our health: no END(S) of the discussion in sight	Review paper
Shahande	2021	Vaping and cardiac disease	Review

h			paper
Larue	2021	Immediate physiological effects of acute electronic cigarette use in humans: A systematic review and meta-analysis	Review paper
Middlekauff	2020	Cardiovascular impact of electronic-cigarette use	Review paper
Orimoloye	2019	Electronic cigarettes and cardiovascular risk: Science, policy and the cost of certainty	Review paper
Jeyakumar	2022	The Effects of E-cigarette Use on Cardiovascular Health: A Systematic Review and Meta-analysis	Review paper
Benowitz	2016	Cardiovascular toxicity of nicotine: Implications for electronic cigarette use	Review paper
Macdonald	2019	Electronic cigarettes and cardiovascular health: What do we know so far?	Review paper
Belkin	2023	Impact of Heated Tobacco Products, E-Cigarettes, and Cigarettes on Inflammation and Endothelial Dysfunction	Only measured acute exposure to e-cigarette
Ben	2023	Pod-based e-cigarettes versus combustible cigarettes: The impact on peripheral and cerebral vascular function and subjective experiences	Only measured acute exposure to e-cigarette
Cossio	2020	Vascular effects of a single bout of electronic cigarette use	Only measured acute exposure to e-cigarette
Mastrangeli	2018	Predictors of oxidative stress and vascular function in an experimental study of tobacco versus electronic cigarettes: A post hoc analysis of the SUR-VAPES 1 Study	Only measured acute exposure to e-cigarette

Kerr	2019	Acute effects of electronic and tobacco cigarettes on vascular and respiratory function in healthy volunteers: A cross-over study	Only measured acute exposure to e-cigarette
Elena Cavarretta	2019	Subjective smoking satisfaction between heat-not-burn, electronic vaping, and traditional tobacco combustion cigarettes: A sub-analysis of the SUR-VAPES 2 trial	Only measured acute exposure to e-cigarette
Rashid	2019	E-cigarette use leads to impaired coronary endothelial function in young adults	Only measured acute exposure to e-cigarette
Rashid	2019	Chronic E-cigarette users demonstrate more persistent coronary endothelial dysfunction than chronic combustible cigarette users	Only measured acute exposure to e-cigarette
Kerr	2017	The immediate effects of electronic cigarette use and tobacco smoking on vascular and respiratory function in healthy volunteers: A crossover study	Only measured acute exposure to e-cigarette
Wolkart	2023	Varied effects of tobacco smoke and e-cigarette vapor suggest that nicotine does not affect endothelium-dependent relaxation and nitric oxide signaling	In vivo/in vitro
Wetherill	2023	Molecular Imaging of Pulmonary Inflammation in Users of Electronic and Combustible Cigarettes: A Pilot Study	In vivo/in vitro
Liu	2023	E-cigarettes Induce Dysregulation of Autophagy Leading to Endothelial Dysfunction in Pulmonary Arterial Hypertension	In vivo/in vitro
Liu	2022	Flavored and Nicotine-Containing E-Cigarettes Induce	In vivo/in

		Impaired Angiogenesis and Diabetic Wound Healing via Increased Endothelial Oxidative Stress and Reduced NO Bioavailability	vitro
Wolkart	2019	Effects of flavoring compounds used in electronic cigarette refill liquids on endothelial and vascular function	In vivo/in vitro
Lee	2019	Modeling Cardiovascular Risks of E-Cigarettes With Human-Induced Pluripotent Stem Cell-Derived Endothelial Cells	In vivo/in vitro
Sivandzade	2019	Assessing the protective effect of rosiglitazone against electronic cigarette/tobacco smoke-induced blood-brain barrier impairment	In vivo/in vitro
Majid	2023	Pod-based e-liquids impair human vascular endothelial cell function	In vivo/in vitro
Dahdah	2022	Immunological Insights into Cigarette Smoking-Induced Cardiovascular Disease Risk	In vivo/in vitro
Mohammadi	2020	Functional impairment of endothelial cells in vitro after exposure to serum from E-cigarette users	In vivo/in vitro
Matheson	2024	Evidence of premature vascular dysfunction in young adults who regularly use e-cigarettes and the impact of usage length	No sole e-cigarette data
Biondi-Zoccai	2020	Cardiovascular Benefits of Switching From Tobacco to Electronic Cigarettes	No sole e-cigarette data
Majid	2022	Effects of Pod-Based Electronic Cigarette Use on Vascular Health and Relation to Volatile Organic Compound Exposure in Young Adults	No sole e-cigarette data
Majid	2022	Association of Volatile Organic Compound Levels With Pod-Based Electronic Cigarette-Induced Changes in Vascular Function of Young Adults	No sole e-cigarette data
Munzel	2020	Effects of tobacco cigarettes, e-cigarettes, and waterpipe smoking on endothelial function and clinical outcomes	No sole e-cigarette data
Jiang	2022	E-cigarette Use and Markers of Endothelial Function in Diverse Young Adults From New York City: The VapeScan Study	No flow-mediated vasodilation data
Davis	2022	Chronic E-Cigarette Exposure Alters Human Alveolar	No flow-

		Macrophage Morphology and Gene Expression	mediated vasodilation data
Traboulsi	2020	Inhalation toxicology of vaping products and implications for pulmonary health	No flow-mediated vasodilation data
Wetherill	2022	Using 18F-NOS PET Imaging to Measure Pulmonary Inflammation in Electronic and Combustible Cigarette Users: A Pilot Study	No flow-mediated vasodilation data
He	2021	Probing early-stage pulmonary pathophysiology in young healthy ecigarettes users using hyperpolarized 129Xe MRI	No flow-mediated vasodilation data
Bandela	2021	Cigarette or E-cigarette content alters autophagy and permeability of lung endothelium	No flow-mediated vasodilation data
Doot	2020	Blood pool selection for quantifying lung inflammation via [18 F]NOS uptake in nicotine users and healthy humans	No flow-mediated vasodilation data
Singh	2020	Biomarkers of inflammation, oxidative stress, pro-resolving lipid mediators, triglycerides, growth factors and tissue injury in electronic cigarette users: Implications for non-invasive assessment of vaping associated lung injuries	No flow-mediated vasodilation data
Tayeb	2017	A cross sectional study reveals an association between electronic cigarette use and myocardial infarction	No flow-mediated vasodilation data
El-Mahdy	2022	Electronic cigarette exposure causes vascular endothelial dysfunction due to NADPH oxidase activation and eNOS	Animal study

		uncoupling	
Spoladore	2023	[The fact about e-cigarettes and cardiovascular risk]	Not written in English

Supplementary Table 4. Detailed information on the flow-mediated vasodilation measurement of selected studies for meta-analysis

Study	Methodologies of flow-mediated vasodilation (FMD) measurement
Haptonstall (2020)	High-resolution ultrasound (Logic 7, General Electric, Inc) measurement of brachial artery FMD and endothelium-independent dilation in response to 0.15 mg sublingual nitroglycerin was performed by the same investigator. Assessments were done with a 7.5-MHz linear array transducer ultrasound system in spectral Doppler mode. A sphygmomanometric cuff was placed just below the antecubital fossa. The brachial artery was imaged with assistance from a probe holder between 5 to 8 cm above the antecubital crease. Image was optimized in B-mode and landmarks were noted and were also marked on the arm to ensure matching images pre/post exposure. Vascular imager software with automated edge-detector was used for recording and analysis (Vascular Analysis Tools, Medical Imaging Applications, LLC). After baseline diameter was recorded for 30 seconds, a sphygmomanometric cuff was inflated to 250mmHg for 5 minutes. The image was recorded 30 seconds before cuff deflation and continued for 2 minutes after release.
Mohammadi (2022)	Vasomotor endothelial function was assessed after 15 minutes of supine rest in a 21°C room, using a standard clinical ultrasound-based method to measure FMD. High-resolution ultrasound of the right brachial artery was performed 1 cm distal to the antecubital fossa with a 10 MHz linear array probe coupled to a GE Vivid 7 Imaging System and Sonosite M-turbo. To assess FMD, after recording baseline B-mode ultrasound images of the brachial artery and spectral Doppler images of flow velocity, a forearm cuff was inflated to 250 mmHg for 5 minutes to induce transient ischemia. Immediately after deflation, Doppler images were obtained to measure reactive hyperemia. Digital images for FMD were analyzed by a blinded investigator with dedicated software (Information Integrity Inc.; Iowa City, Iowa) and Doppler velocity signal with NIH ImageJ. To minimize the variation in FMD during the ovarian cycle, menstruating women were tested during the first 5 days of their menstrual period.
Fetterman (2020)	Brachial artery diameter was measured at baseline and after a 5-minute occlusion (blood pressure cuff attached to the lower arm inflated to 200 or 50 mm Hg higher than the systolic pressure) to determine flow-mediated dilation, a noninvasive measure of conduit artery endothelial-dependent vasodilation. Resting and hyperemic flow velocities and shear stress were measured in the brachial artery using Doppler ultrasound. All vascular images were analyzed at Boston University using Vascular

	Research Tools Brachial Analyzer for Research V.6.8.5 (Medical Imaging Applications, LLC) by a technician blinded to tobacco product use group.
Boakye (2023)	FMD was assessed non-invasively using high-resolution brachial artery ultrasound probe (Toshiba Aplio; Ultrasound transducer PLT-1202S 12MHz). Brachial FMD (imaged 2 cm above antecubital fossa; forearm occlusion; cuff pressure to 250 mmHg; occlusion for 5 mins) was assessed by the same experienced technician for all participants.

The information was extracted from the selected papers for meta-analysis.

Supplementary Table 5. Risk of bias assessment for selected studies for meta-analysis- Modified New Castle Ottawa scale (Cross-sectional study)

Study	Selection				Comparability	Outcome		Score
	Representativeness of the sample	Sample size	Non-respondents	Ascertainment of exposure (max**)	Confounding controlled (max**)	Assessment of the outcome (max**)	Statistical test	Total
Haptonstall (2020)				*	**	**	*	6/10
Mohammadi (2022)				*	**	**	*	6/10
Fetterman (2020)		*		*	**	**	*	7/10
Boakye (2023)				*	**	**	*	6/10

Supplementary Figure 1. Funnel plot for evaluating the publication bias of pooled mean differences of flow mediated vasodilation derived from selected studies (exclusive e-cigarette use vs. non-use)

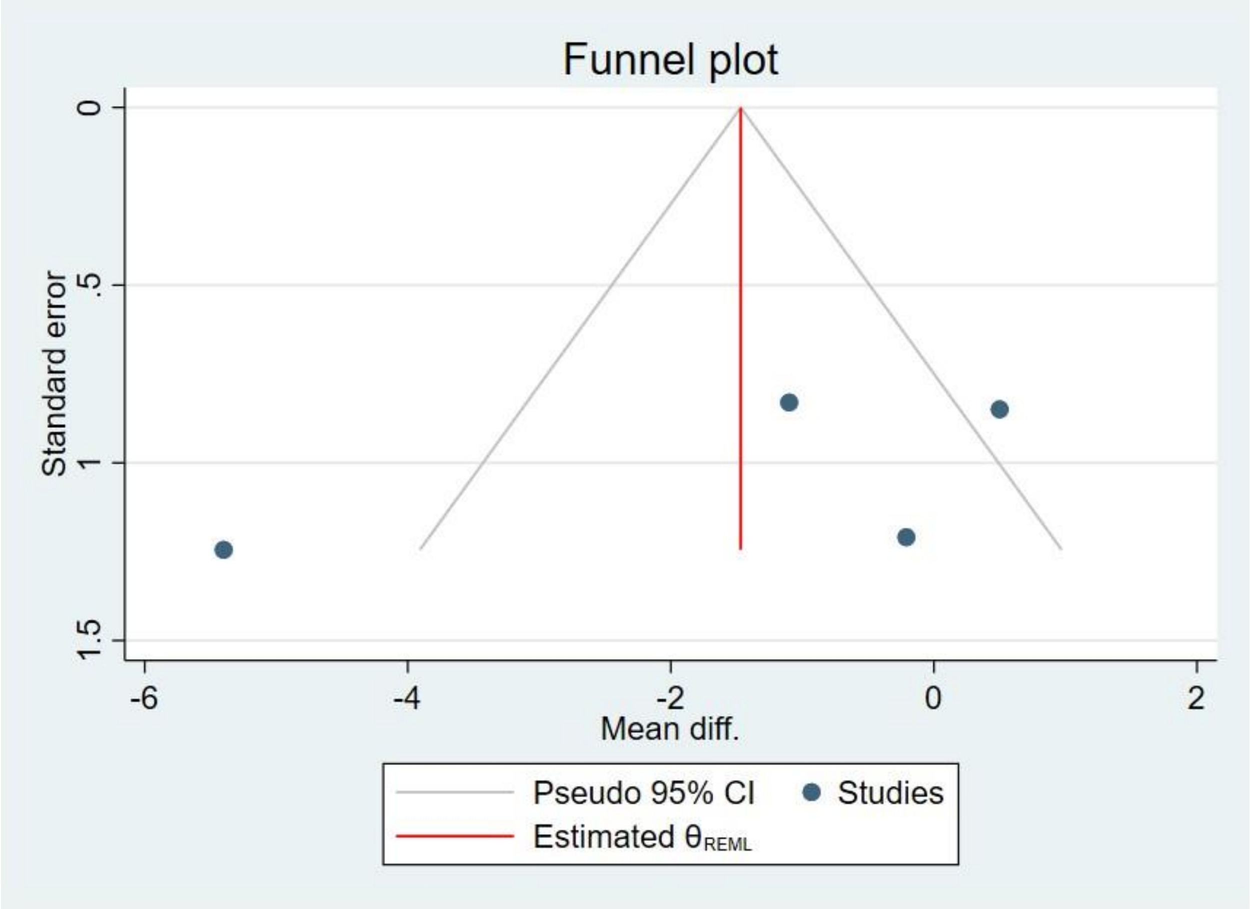

* x-axis: mean difference of flow mediated vasodilation (exclusive e-cigarette use vs. non-use); y-axis: standard error of mean difference. A random-effects model was employed.

Supplementary Figure 2. Funnel plot for evaluating the publication bias of pooled mean differences of flow mediated vasodilation derived from selected studies (exclusive e-cigarette use vs. exclusive combustible cigarette use)

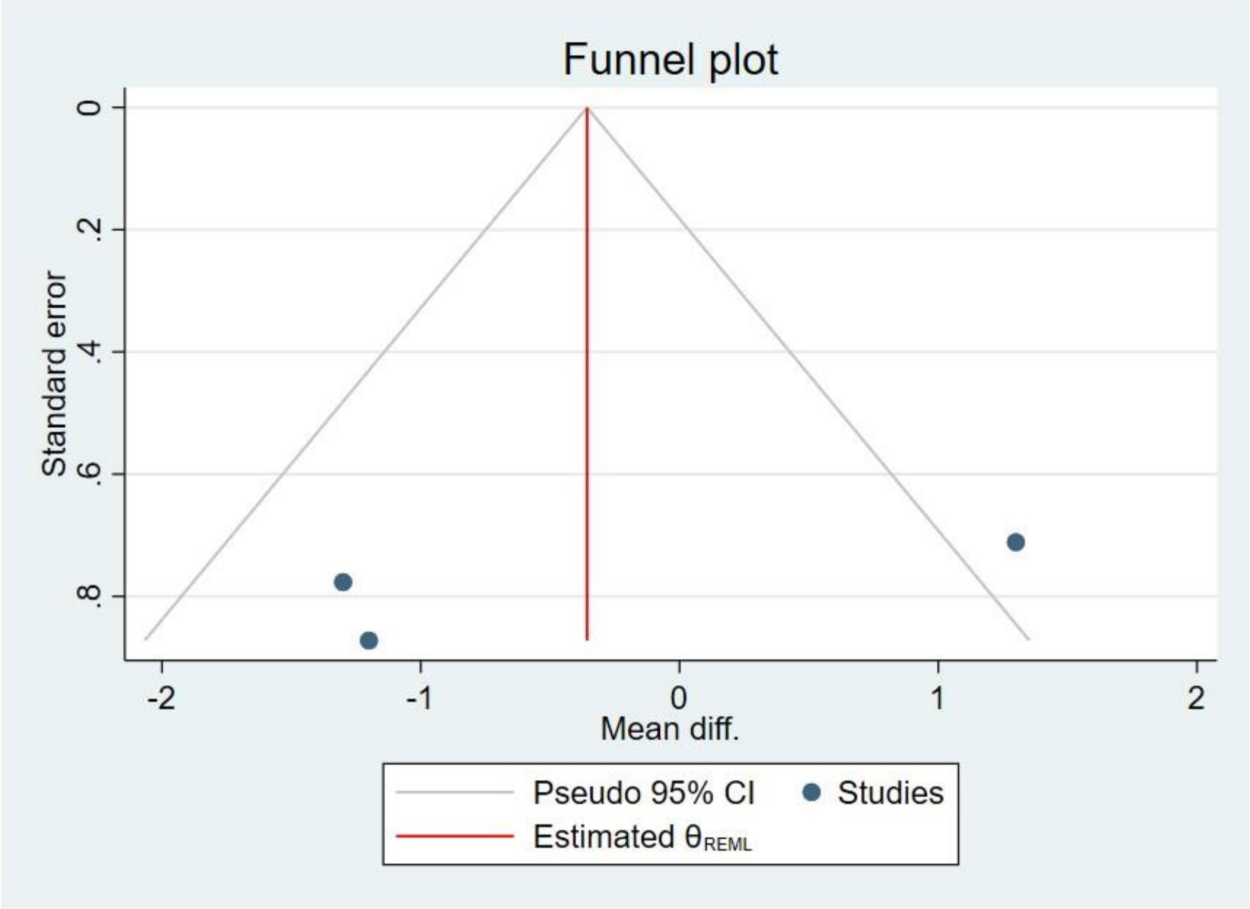

* x-axis: mean difference of flow mediated vasodilation (exclusive e-cigarette use vs. exclusive combustible cigarette use); y-axis: standard error of mean difference. A random-effects model was employed.

Supplementary Figure 3. Funnel plot for evaluating the publication bias of pooled mean differences of flow mediated vasodilation derived from selected studies (exclusive combustible cigarette use vs. non-use)

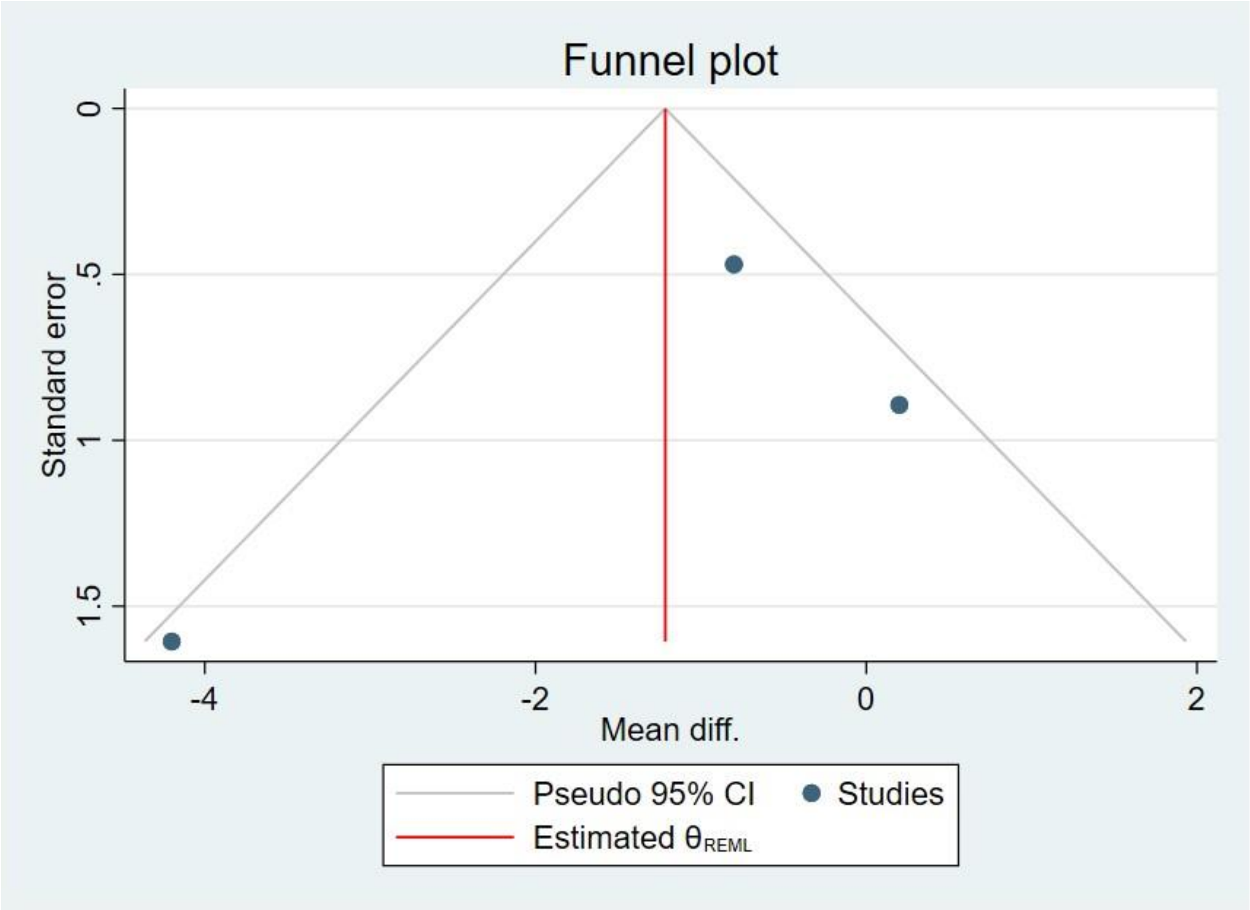

* x-axis: mean difference of flow mediated vasodilation (exclusive combustible cigarette use vs. non-use); y-axis: standard error of mean difference. A random-effects model was employed.

©2024 Lee J. et al.
